# Acupuncture is effective in the treatment of postprandial distress syndrome: A systematic review and meta-analysis

**DOI:** 10.1097/MD.0000000000033968

**Published:** 2023-06-23

**Authors:** Ganchen Xiao, Yingtao Zhao, Xingyu Chen, Fangli Xiong

**Affiliations:** a First Affiliated Hospital of Guizhou University of Traditional Chinese Medicine, Guizhou University of Traditional Chinese Medicine, Guiyang, China; b Graduate School of Guizhou University of Traditional Chinese Medicine, Guizhou University of Traditional Chinese Medicine, Guiyang, China.

**Keywords:** acupuncture, dyspepsia, functional dyspepsia, meta-analysis, PDS, syndrome

## Abstract

Traditional Chinese medicine advocates the use of acupuncture for the treatment of postprandial distress syndrome (PDS) in people with Functional dyspepsia, but large clinical trials of acupuncture have produced controversial results. This study aims to confirm the clinical significance of acupuncture in the treatment of PDS .

**Methods:**

This study only randomized controlled trials were included from the following databases: CNKI, Medline, Cochrane Central, Web of Science, and Clinical Trial. The risk of bias in the included studies was assessed using Revman 5.4.1 (Revman 2020), and all 12 included studies were considered to have a low risk of bias. This study used Stata 16.1 for data analysis, including sensitivity analysis and publication bias test. The quality of each study was evaluated with the Cochrane tool. The main outcomes included the overall therapeutic rate, the SID score, the HADS Score, The NDI score, and Side effects.

**Results:**

This study identified a total of 1532 studies interested in the curative effect of acupuncture on Postprandial discomfort syndrome (PDS) and finally included a total of 12 studies with 1113 patients after identifying their abstracts, titles, and full text. The process of literature searches and identifying is shown in Figure 1 and data analysis showed that acupuncture is effective in the treatment of PDS and promotes the life quality of patients.

**Conclusions:**

This study analyzed the effects of acupuncture on PDS from 5 aspects: overall therapeutic rate, SID, HADS, NDI, and side effects, overall therapeutic rate as primary outcome measure. Statistical analysis results showed that acupuncture has a significant effect on the treatment of PDS. In conclusion, it is an effective clinical treatment method. Also, the potential bias in the included studies, high-quality studies are needed to further confirm the possible side effects of acupuncture in treatment.

## 1. Introduction

### 1.1. Background and available evidence

Functional dyspepsia (FD) is a group of clinical syndromes caused by gastroduodenal dysfunction.^[1,2]^ Upper gastrointestinal endoscopy excludes organic diseases that cause dyspepsia symptoms and seriously affect the daily activities of patients. According to Rome IV standard, functional dyspepsia is divided into 2 subtypes of postprandial distress syndrome (PDS) and upper abdominal pain syndrome.^[3,4]^ The symptoms of patients with PDS are related to eating, and the clinical manifestations are postprandial fullness, early fullness, and upper abdominal distension.^[5]^ Epidemiological survey results show that the global prevalence of functional dyspepsia is about 15%, of which nearly 2/3 of patients meet the diagnosis of PDS. PDS has a considerable impact on patients’ quality of life, health care expenditure, and daily activities including work. Due to the long course of the disease, the curative effect of drug treatment is limited, and it is easy to relapse.^[5]^ It is easy to induce or even aggravate negative emotions such as depression and anxiety in some patients.^[6]^

One treatment modality that has gained increasing attention in recent years is acupuncture. Acupuncture is a traditional Chinese medicine technique that involves inserting thin needles into specific points on the body to stimulate and balance the flow of energy. Acupuncture has been used for thousands of years to treat a wide range of conditions, including digestive disorders. At present, meta-analyses on acupuncture treatment of PDS are very few and cannot reach an obvious conclusion,^[7]^ so this study is to further confirm the efficacy and safety of acupuncture treatment of PDS. In this study, the patients with spleen-stomach qi deficiency syndrome of PDS in the multicenter randomized controlled clinical trials were retrospectively analyzed to evaluate the clinical symptoms, quality of life, and the efficacy of acupuncture in the treatment of patients with spleen-stomach qi deficiency syndrome of PDS.^[8]^ The specific reports are as follows.

Clinically, traditional Chinese medicine, acupuncture, acupoint injection, massage, acupoint application, and other treatment methods are adopted, among which acupuncture is the most widely used and the curative effect is more remarkable.^[9–11]^ Studies have reported that acupuncture can treat functional dyspepsia and improve mood disorders.^[12,13]^ However, the current clinical acupuncture treatment is not standardized, and there is a lack of a unified standard for acupoint selection. To further explore the efficacy of acupuncture in the treatment of PDS FD, this study provides reliable theoretical support for the treatment of PDS FD with different acupoint acupuncture schemes in clinic. Through clinical research, our research group found that acupuncture can significantly improve the overall symptoms of PDS patients and improve the elimination rate of symptoms.

### 1.2. Value of this review

PDS can seriously affect the quality of life, increase medical expenses and lead to mental disorders such as anxiety and depression. This study provides an economic and reliable method for the treatment of PDS, which can significantly improve the quality of life of patients.

## 2. Method

The reporting of this systematic review was guided by the standards of the Preferred Reporting Items for Systematic Review and Meta-Analysis Statement.

All trials concerning the curative effect of acupuncture on PDS were selected from the Embase, Cochrane Central Register of Controlled Trials, PubMed, CNKI, and the Chinese Biomedical Literature Database. The criteria for excluding the literature are as follows: The study did not carry out in clinical randomized controlled trials; Participants or outcomes and interventions do not meet the inclusion criteria; Enrollment of patients with organic digestive diseases; Incomplete or unavailable data; Any conference papers, reviews, case reports, experience summaries, and repeated literature (only the earliest one is retained in the papers published in multiple languages) were rejected. Appendix 1 (Supplemental digital content, http://links.lww.com/MD/J104) gives the search strategy.

This Study summarized the curative effect of acupuncture on PDS in the following measures: Overall effective rates; The SID score; The HADS Score (a score for patients’ quality); The NDI score; and Side effects. The overall effects were summarized in Figure 2A–E. The Overall therapeutic rate as the ratio of PDS symptoms to remission after treatment, and each of the included trials was carefully reviewed and counted, and according to the different criteria of each trial, counted the number of people in remission of PDS after treatment.

**Figure 1.**
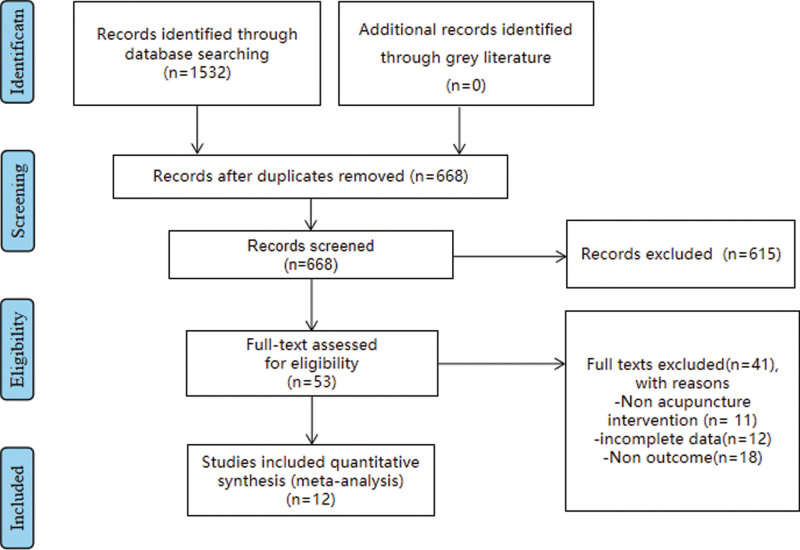
The flow chart.

Data statistics, tests for heterogeneity, and sensitivity analysis, and publication bias by STATA 16.1 (STATA 2020). Both egger’s test, funnel plots, and Begg’s test for each outcome (Figure 3A–E) and the forest plots for each outcome measure by Revman 5.4.1 (Revman 2020). Quality evaluation was generated by Revman 5.4.1 (Revman 2020) in the following assessments: Random sequence generation; Allocation concealment; Blinding of participants and personnel; Blinding of outcome assessment; Incomplete outcome data; Selective reporting; and Other bias. A summary of the risk of bias was provided in Figure 4. Also, *I*^2^ is a statistical measure used to assess the degree of heterogeneity among studies included in a meta-analysis. It ranges from 0% to 100% and measures the proportion of total variability due to between-study heterogeneity. If *I*^2^ is greater than 50%, it indicates a substantial level of heterogeneity and a random-effects model should be used for data analysis. Otherwise, a fixed-effects model can be used. This study defined that the heterogeneity is high when *I*^2^ is greater than 30%.

**Figure 2.**
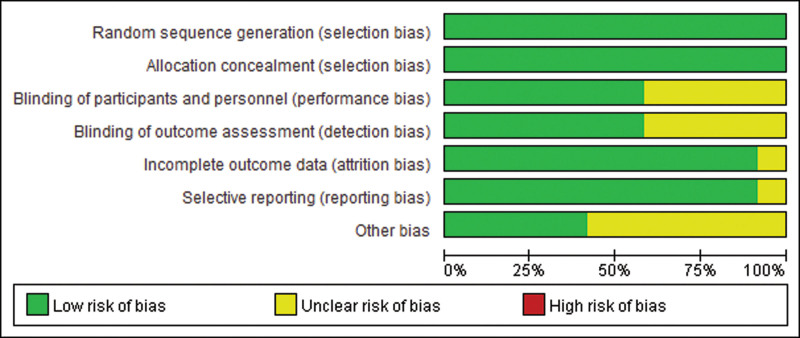
(A) Overall therapeutic rate chart. (B) The SID score. (C) The HADS score. (D) The NDI score. (E) Side effects. HADS = hospital anxiety and depression scale, NDI = Nepean dyspepsia index, SID = symptom index of dyspepsia.

**Figure 3.**
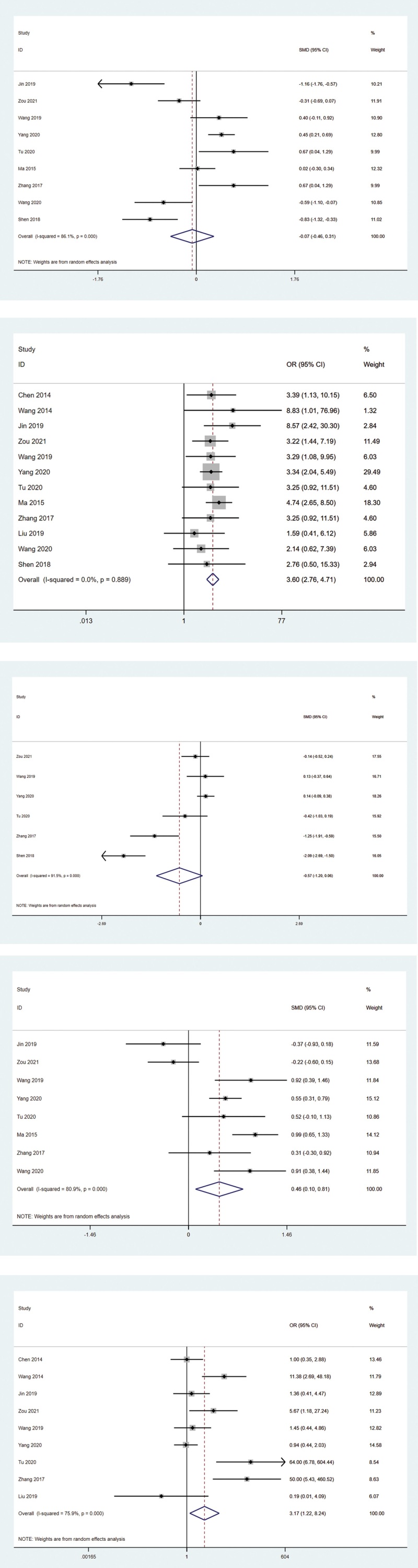
Funnel plots of each outcome: A. Overall effective rate. B. The SID score. C. The HADS score. D. The NDI score. E. Side effects. HADS = hospital anxiety and depression scale, NDI = Nepean dyspepsia index, SID = symptom index of dyspepsia.

**Figure 4.**
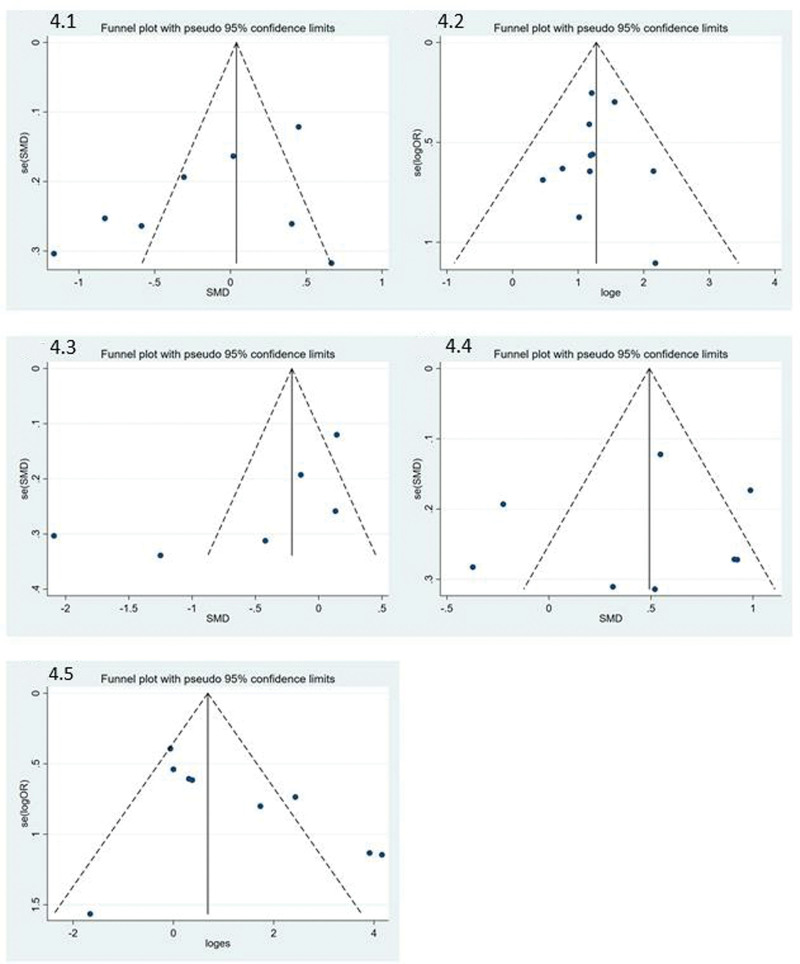
Risk of bias.

Three researchers (G.X., Y.Z., and X.C.) independently conducted the view of titles and abstracts and the full-text review. study exclusion and the inclusion based on their own exclusion criteria. And 2 reviews (G.X. and Y.Z.) extracted the following studies: basic of patients, outcomes measures. Adverse reactions, et al, quality assessments of studies were reported to the Cochrane Systematic Evaluator’s Manual. Any disagreement was reported to an experienced doctor (X.C.). Another researcher (X.C.) double-checked the consistency of the statistical results transformed into the software with the data provided in the original literature to avoid any misinputs. One reviewer (G.X.) performed statistical analysis by STATA 16.1 to obtain the results of each measure and conduct a recheck.

## 3. Result

This study identified a total of 1532 studies associated with the efficacy of acupuncture for PDS, from which finally included a total of 12 studies of 1113 patients published in papers or on the website from July 20, 1989 to August 3, 2021. Any studies that did not meet the criteria were excluded. A flow chart for the progress of literature retrieval and the inclusion or exclusion of studies in Figure 1.

Three Reviewers (G.X., Y.Z., and X.C.) independently recorded the following characteristics of 12 studies: Mean age, Course of disease, intervention measures, Intervention group, Frequency and treatment course et al, and more details were shown in Table 1 (Characteristic of studies).

**Table 1:** Characteristics of studies.

Studies (year)	Diagnostic criteria	Patient admission time	No. of participants (T/C) (M/F)	Mean age (yr) (T/C)	Course of disease (T/C)	Intervention group	Frequency and treatment course	Control group	Outcome
Chen 2014	Rome III	From February 2011 to November 2013	57/57 60/54	68.39 ± 9.67	NS	Verum Acupuncture	Once daily 7/wk for 4 wk	Gasmotin	OTE, gastric emptying time, gastric motilin levels
Wang 2014	Rome III	From October 2010 to October 2012	30/30 37/23	(68.2 ± 9.1)/(67.8 ± 8.5)	NS	Verum Acupuncture	Once daily 5/wk for 4 wk	Gasmotin	OTE, gastric emptying time, gastric motilin levels
Jin 2019	Rome IV	From October 2017 to October 2018	26/25 14/37	(43 ± 17)/(41 ± 16)	(42.7 ± 54.1)/(59.9 ± 65.9)	Verum Acupuncture	Every other day, 3/wk for 4 wk	Sham Acupuncture	OTE, SID, NDI, adverse
Zou 2021	Rome IV	From April 2017 to January 2019	55/53 35/73	(44.4 ± 13.2)/(37.3 ± 11.5)	(42.69 ± 54.07) mo/(59.92 ± 65.88)	Verum Acupuncture	Every other day, 3/wk for 4 wk	Sham Acupuncture	OTE, SID, NDI, HADS, adverse
Wang 2019	Rome IV	From October 2018 to January 2019	30/30 38/22	(42.3 ± 12.8)/(40.8 ± 10.3)	(48 (18, 78) mo/36 (19.5,72)	acupuncture treatment 3/w	Every other day, 3/wk for 4 wk	Acupuncture treatment 1/wk	OTE, SID, NDI, HADS, adverse
Yang 2020	Rome IV	From April 2017 to January 2019	138/140 91/187	41.6 (13.1)/ 41.2 (13.1)	57.3 (63.7)/61.6 (64.4)	Verum Acupuncture	Every other day, 3/wk for 4 wk	Sham Acupuncture	OTE, SID, NDI, HADS, adverse
Tu 2020	Rome IV	From July 2016 to November 2016	21/21 14/28	(44.8 ± 13.3)/(46.0 ± 13.2)	36 [24, 60]/54 [24, 120]	Verum Acupuncture	Every other day, 3/wk for 4 wk	Sham acupuncture	OTE, SID, NDI, HADS, adverse
Ma 2015	Rome III	From April 2008 to October 2009	79/71 46/104	(38.8 ± 13.8)/(36.0 ± 13.0)	(75.7 ± 74.5) mo/ (67.3 ± 80.3)	Verum Acupuncture	Every other day, 5/wk for 4 wk	Sham acupuncture	OTE, SID, NDI
Ma 2016	Rome III	From July 2016 to September 2016	21/21 14/28	(44.8 ± 13.3)/(46.0 ± 13.2)	(67.7 ± 87.1) mo/(68.0 ± 52.7)	Verum Acupuncture	Every other day, 3/wk for 4 wk	Sham Acupuncture	OTE, SID, NDI, HADS, adverse
Ma 2017	Rome IV	From January 2017 to June 2018	40/40 29/51	(37.98 ± 11.18)/(39.83 ± 12.38)	(22.63 ± 9.92) mo/(25.58 ± 11.86)	acupuncture	Every other day, 1/2 wk for 8 wk	Moroxyline	OTE, SF-36, adverse
Ma 2018	Rome IV	From January 2019 to November 2019	30/30 28/32	(42.40 ± 10.15)/(41.30 ± 10.72)	(14.68 ± 5.51) mo/(14.13 ± 4.99)	Electronic moxibustion	Once daily 7/wk for 2 wk	Mosapril Citrate Tablets	OTE, SID, NDI, adverse
Ma 2019	Rome IV	From May 2017 to February 2018	34/34 55/13	(45.71 ± 11.256)/(44.21 ± 11.591)	(18.38 ± 10.216) mo/(18.56 ± 11.076)	Electronic moxibustion	Once daily 5/wk for 4 wk	Moroxyline	OTE, SID, HADS

HADS = hospital anxiety and depression scale, NDI = Nepean dyspepsia index, SID = symptom index of dyspepsia.

The risk of bias assessment indicated that all the included studies had a low risk of bias. Despite the potential for bias, the overall findings of the meta-analysis were consistent across studies. All the trials included in this study, acupuncture points included Baihui (DU20), Danzhong (RN17) and Zhongwan(RN12), Qi Sea (RN6), bilateral Tianshu (ST25), Neiguan (PC6), Zusanli (ST36), Gongsun (SP4). All the acupuncture points are based on World Health Organization standard acupuncture sites and exhibitions where all needles are inserted for at least 30 seconds Reach de qi (a sense of composition, including Pain, numbness, swelling, and heaviness). It is considered to be an important part of the efficacy of acupuncture.

### 3.1. Test for overall effect

#### 3.1.1. Overall therapeutic rate.

A total of 1113 patients were enrolled in 12 studies, which provide the effect of acupuncture on the overall effective rate in patients with PDS. All the studies were intragroup-controlled trials with a simple population (Chinese) and male patients mostly. Heterogeneity test for the included studies was done by STATA 16.1, and results showed *P* = .889 and *I*^2^ = 0.0%, a very low heterogeneity, so a random fixed effects model is used, which suggests it’s the evidence of high quality. The result of data analyses showed that acupuncture is effective in the treatment of PDS (OR 3.60, 95% CI [2.76, 4.71]), which suggest the significant therapeutical effect of acupuncture. The results were summarized in Figure 2A.

#### 3.1.2. The SID score.

There were 9 studies including 425 patients associated with the effect of acupuncture on the HAS score change in patients with PDS. 9 studies were all intragroup-controlled trials of a simple population (Chinese) and male patients mainly. Heterogeneity in these 9 studies was test using STATA 16.1, and the results showed that *P* < .001, *I*^2^ = 86.1%, indicating high heterogeneity, for which a random effect model is needed. The sensitivity analysis of these 9 studies was performed with STATA 16.1 and found possible sources of heterogeneity (Jin 2019), which may be related to specific techniques of acupuncture. The pooled data showed that acupuncture had no significant effect on SID score in PDS patients (SMD −0.07, 95% CI [−0.46, 0.31]). Revman5.4.1 (Revman 2020) provided forest plot for each outcome measure. The results were summarized in Figure 2B.

#### 3.1.3. The HADS Score (a score for patients’ quality).

Six studies enrolled 598 patients, all of which were related to the effectiveness of acupuncture on HADS score in patients with PDS. All studies were within-group controlled trials with a simple population (Chinese) and male patients mostly. The heterogeneity test included in the study was carried out by STATA 16.1, where *P* < .001, *I*^2^ = 91.5%, it shows very high heterogeneity, for which a random effects model must be used. The sensitivity analysis of these 6 studies was done by STATA 16.1 and found possible sources of heterogeneity (Shen 2018), which may be related to specific techniques of acupuncture. Data analysis showed that acupuncture does not have significantly effective on HADS score of PDS (SMD −0.57, 95 % CI [−1.20, 0.06]). These results are summarized in Figure 2C.

#### 3.1.4. The NDI score.

Eight studies with 791 patients enrolled focused on the effect of acupuncture on the NDI score in patients with PDS. The 8 studies were all intragroup-controlled trials with a simple population (Chinese) and male patients mostly. The heterogeneity test included in the study was carried out by STATA 16.1, in which *P* < .001 and *I*^2^ = 80.9%, it shows very high heterogeneity, for which a random effects model must be used. The sensitivity analysis of these 8 studies was done by STATA 16.1 and found possible sources of heterogeneity (Jin 2019), which may be related to specific techniques of acupuncture. The pooled data showed that acupuncture can increase the NDI score of PDS patients (SMD 0.46, 95% CI [0.10, 0.81]), which suggests acupuncture can increase the NDI score. The results were summarized in Figure 2D.

#### 3.1.5. Side effects.

There were 10 studies with 448 patients in the experimental group and 717 in the control group interested in the effect of acupuncture on the Side effects in patients with PDS. And the 10 studies were all intragroup-controlled trials with a simple population (Chinese) and male patients mostly. Heterogeneity in these 10 studies was test using STATA 16.1, and the results showed that *P* < .001, *I*^2^ = 72.9%, indicating high heterogeneity, for which a random effect model is needed. And this study underwent sensitivity analysis for the 4 studies by STATA 16.1, the results showed an equal effect of each study. After analysis, the variety of populations to be the possible source of heterogeneity. The pooled data showed that acupuncture can increase the rate of side effects of PDS patients (OR 3.17, 95% CI [1.22, 8.24]). The results were summarized in Figure 2E.

## 4. Discussion

Functional dyspepsia (FD) is a common disease characterized by multiple upper gastrointestinal symptoms, which can negatively affect the quality of life of patients.^[4,14]^ Rome III consensus defined functional dyspepsia as symptoms in the gastroduodenal region without any organic, systemic or metabolic disease that might explain symptoms.^[15,16]^ It has grouped functional dyspepsia into PDS characterized by widespread eating-related early satiety; epigastric pain syndrome, characterized by epigastric pain, is generally not associated with eating.^[17]^

Previous studies have shown that PDS patients are more than epigastric pain syndrome patients: in the general population, about half of the subjects with dyspepsia symptoms report their complaints after a meal. The study also reported that the highest intensity of postprandial symptoms was postprandial fullness in people who reported symptoms related to diet, while upper abdominal pain in people who reported symptoms unrelated to diet.^[18]^

Traditionally, FD, especially PDS, is related to gastric motility and gastric regulation dysfunction. However, studies have shown that gastric physiological disorder is not related to symptoms or severity of symptoms.^[19,20]^ Emerging evidence has shifted the focus from changing gastric motility to brain-gut communication. Compared with healthy people, FD patients had changes in brain functional connectivity. A previous study has shown that acupuncture can significantly reduce glucose metabolism in the key areas of brain-gut communication-insula, precingulate cortex, and hypothalamus. The inactivation of these areas is related to the relief of dyspepsia symptoms.^[21,22]^

Acupuncture originated in China and has been practiced for more than 3000 years, and was introduced into Europe and the United States since the 16th century.^[23]^ Acupuncture research began in the eighteenth century and has developed rapidly since then. In the past, doctors tried to apply acupuncture to clinical practice, while scientists focused on the possible characteristics of acupoints and meridians. In modern times, scientists have been trying to evaluate the real efficacy of acupuncture and the potential physiological and biological mechanisms of acupuncture.^[8]^

Acupuncture is widely accepted as an effective treatment option for gastrointestinal diseases in clinical practice. Several studies have shown that acupuncture can promote gastrointestinal motility. However, the efficacy of acupuncture for FD has not been proven. To our knowledge, few studies have specifically designed acupuncture for PDS.

This study have included 5 outcome measures from twelve studies and 1113 patients in this review, these was results after statistical analysis: acupuncture is effective in the treatment of PDS (OR 3.60, 95% CI [2.76, 4.71]); has no significant effect on SID score (SMD −0.07, 95% CI [−0.46, 0.31]) and HADS score (SMD −0.57, 95% CI [−1.20, 0.06]) in PDS patients; acupuncture can increase the NDI score of PDS patients (SMD 0.46, 95% CI [0.10, 0.81]); incidence of side effects of PDS patients is increased (OR 3.17, 95% CI [1.22, 8.24]). Finally, the concluded that acupuncture has a significant therapeutic effect on PDS: acupuncture can alleviate the symptoms of PDS, improve the symptom of dyspepsia. And the quality of patients’ daily life was improved, which may be related to the changes in the patients’ perception and their subjective feelings.

This study defined the heterogeneity is high when I-squared is greater than 30%, which is lower than the heterogeneity measured in this study generally. The possible sources of this heterogeneity may include the following items: Different research teams have great differences in acupuncture point selection, point selection strength, and point selection technology. The age and sex ratios of the included population are quite different. Subjective differences can exist when different researchers evaluate scores. The heterogeneity of SID score measurement is 86.1%, which may be due to the small number of studies included.

Finally previous meta-analysis on acupuncture treatment for PDS were scarce. This study aims to further confirm the effective of acupuncture treatment for PDS and provide guidance for clinical work. The results of this study show that acupuncture is effective in treating PDS, in addition, previous studies rarely mentioned the side effects of acupuncture in the treatment of PDS. The results of this study show that acupuncture can cause some side effects to patients, so clinicians need to use acupuncture according to the actual situation.

## 5. Limitations

There are still some limitations in this review: The sample size of included studies is small and more studies are needed to provide more evidence. The population included in this review is relatively single (all Chinese), which may lead to one-sided conclusions. There is no stratified and random description according to the characteristics of patients, which may lead to bias in the efficacy evaluation. Different studies have different acupuncture treatment methods and acupoint selection methods for patients, which to some extent affects the efficacy evaluation of the research results. The incidence of PDS is affected by family environment, social and psychological factors, and PDS is recurrent and persistent. However, the included literature has a short course of treatment and a few follow-up visits, which cannot indicate the efficacy of long-term follow-up.

## Author contributions

**Conceptualization:** Ganchen Xiao, Yingtao Zhao, Xingyu Chen.

**Data curation:** Xingyu Chen.

**Formal analysis:** Ganchen Xiao, Xingyu Chen.

**Investigation:** Yingtao Zhao, Xingyu Chen.

**Methodology:** Ganchen Xiao, Yingtao Zhao.

**Project administration:** Ganchen Xiao.

**Resources:** Ganchen Xiao.

**Software:** Ganchen Xiao.

**Supervision:** Ganchen Xiao, Fangli Xiong.

**Validation:** Fangli Xiong.

**Visualization:** Ganchen Xiao.

**Writing – original draft:** Ganchen Xiao.

**Writing – review & editing:** Fangli Xiong.

## Supplementary Material


