# Supplementary material for: Acupuncture is effective in the treatment of postprandial distress syndrome: A systematic review and meta-analysis
Source: Medicine (Baltimore). 2023 Jun 23;102(25):e33968. doi: 10.1097/MD.0000000000033968 (PMC10289664; doi:10.1097/MD.0000000000033968)
Supplement: Supplementary file 1 [file medi-102-e33968-s001.pdf]

Appendix 1. The search strategy

	PUBMED
SEARCH STRATEGY	
**NUMBER**	Items
**#1**	Acupuncture【Mesh】
**#2**	needle【Title/Abstract】
**#3**	electroacupuncture 【Title/Abstract】
**#4**	fire needle【Title/Abstract】
**#5**	needle warming moxibustion【Title/Abstract】
**#6**	acupoint catgut embedding 【Title/Abstract】
**#7**	#1 OR #2 OR #3 OR #4 OR #5 OR#6
**#8**	Postprandial distress syndrome【Mesh】
**#9**	#7 AND #8
